# Early childhood educator perceptions of risky play in an outdoor loose parts intervention

**DOI:** 10.3934/publichealth.2021017

**Published:** 2021-03-08

**Authors:** Rebecca A Spencer, Nila Joshi, Karina Branje, Naomi Murray, Sara FL Kirk, Michelle R Stone

**Affiliations:** 1 School of Health & Human Performance, Dalhousie University, 6230 South St, PO BOX 15000, Halifax, NS, Canada; 2 Healthy Populations Institute, Dalhousie University, 1318 Robie St, PO BOX 15000, Halifax, NS, Canada

**Keywords:** early childhood, education, loose parts, risky play, educator perspectives

## Abstract

Free play is important in early childhood and offers physical and mental health benefits. Outdoor play offers opportunity for children to use natural elements and promotes physical activity, among other health benefits, including exploring their environment and taking risks. Risky outdoor play may involve challenges, heights, speed, and the potential for injury, but has been associated with increased physical activity levels, decreased sedentary behaviour, improved mental health, and social benefits. The integration of loose parts, or open-ended, unstructured materials, into play environments, has been associated with positive social behaviours, creativity, and improved problem-solving, confidence, and resilience. As opportunities for risky play in early childhood are determined by adults, including early childhood educators, it is important to understand their perspectives on these types of play. The purpose of this study was to explore early childhood educators' perspectives of risky play, in the context of the Physical Literacy in the Early Years (PLEY) intervention. PLEY was a mixed methods study that aimed to evaluate a loose parts intervention in early childcare settings. This paper used Qualitative Description to explore educators' perspectives. Data were collected from 15 focus groups with early childhood educators. Four themes were identified through thematic analysis. The first explains how risky play with loose parts contributes to evolution in educator perceptions; the second describes how educators' perceptions of risk are connected to institutions and systems; the third illustrates how educators developed strategies to facilitate risky play with loose parts; and the fourth demonstrates how educators perceive risky play as beneficial for children's healthy development. This project highlights societal shifts in play and how loose parts and risky play fit into the ongoing evolution in play, from the perspectives of early childhood educators.

## 1. Introduction

Unstructured, self-directed, free play dominates early childhood and affords children numerous physical, cognitive, and mental health benefits [1]. The outdoor environment offers particular play opportunities and health benefits for children that cannot be replicated indoors [2]. When children play outdoors, they are able to move freely, explore new movements, and exert more energy; they have fewer restrictions in space and more opportunity to use natural elements [1],[3],[4]. Children are happier, experience lower levels of anxiety, and have more energy when they play outdoors [5],[6]; physical activity levels are also higher, and sedentary behaviour lower, when children are outside compared to inside [4]. Importantly, when children are given the opportunity to play outdoors, they are able to explore their environment and take risks [5],[7] which is critical for healthy child development [6].

Risky play is described as thrilling and exciting play, that may involve challenges, heights, speed, tools, rough and tumble play, and testing limits, with the possibility of physical injury [6],[8],[9]. Children have an evolutionary need to engage in free, unstructured, exploratory play outdoors [10]. Increasingly, research is showcasing the benefits and importance of risky outdoor play [1],[4],[6],[11]. A review by Brussoni et al. (2015) determined that risky outdoor play has many positive effects on health, including increased physical activity and decreased sedentary behaviour, as well as improved learning, mental and physical health, and increased play time, social interaction and creativity [6]. Through risky play, children have opportunity to be physically active [12], become more independent, confident, and resilient [5], and learn important self-regulation skills [1]. Researchers have recently suggested that engagement in risky play can offer opportunity for children to navigate uncertainty and coping, leading to decreased anxiety over time [13].

While the health, social, and developmental benefits of risky play are becoming understood, there has been historical focus on its potential dangers as well. While risky play is inherently, of course, risky [14], a recent systematic review indicates that although there has been a focus on danger, the vast majority of risky play incidents are minor [6]. Belief in benefits of risk taking need not lead to complacency in safety, but requires distinguishing between appropriate well-managed risk and danger or hazards [15]. While restrictive risk-reduction strategies might ensure momentary child safety, they may also impede their healthy development [11],[14]. Children learn from experience, even of injury, and are often able to assess their own risk [14]. Risky play allows children to engage in risk assessment, negotiate risk, and understand their limits [11]. Historically, however, risk has been narrowly defined, with negative connotations, which has contributed to risk-aversive practices and a decline in opportunities for children to engage with risk [1],[11],[16].

The Canadian Position Statement on Active, Outdoor Play states, “access to active play in nature and outdoors - with its risks - is essential for healthy child development”, highlighting both the importance of risky play, and the outdoor context as space for facilitating risky play [1]. Outdoor play lends itself to more risky and adventurous play [13]. Children today spend less time outdoors than their parents did, and more time in institutions and structured activities [1],[17]. Concerns about safety have limited children's access to risky outdoor play and independent mobility [18],[19]. Further, children's access to outdoor spaces, with their risks, may be limited by beliefs that children lack the competence to engage with the world alone and are in danger when outside [20]. As a result, it is becoming increasingly difficult for children to have the opportunity to engage in unstructured, risky, outdoor play.

Risk-taking opportunities in early childhood are also largely influenced by adults, including educators [12],[13],[16]. Due to increased emphasis on both safety and school readiness, many early years programs are focusing more on structured activities, and educators are acknowledging ideas around risky play are shifting and evolving [11],[21]. While early learning environments have been identified as particularly important contexts for children to be able to learn about and engage with risk, navigating risky play in these environments is a complex and multi-faceted issue [11],[12],[14],[17],[22],[23]. Research exploring educators' perceptions of risky play suggests that early childhood educators recognize the importance of, but identify barriers, including their understanding of safety, regulations, accountability, and potential litigation, with educators in identified less-litigious contexts being more open to risky play [12],[15],[16],[22]–[26]. Research has begun to identify strategies employed by educators in negotiating risky play, including supervision and observation [6],[11],[14],[15],[22],[27], though further investigation is warranted to support early childhood educators in navigating this complex issue.

There is overlap between risky play, outdoor play, physical activity, and learning environments [13]. Features of the early learning environment influence the extent of available risky play opportunities [28]. Previous research highlights the importance of environments that support children to explore, experiment, accept challenges, and take risks [6],[11],[15],[22]. The integration of loose parts into children's outdoor play environments may facilitate opportunities for risky play. Loose parts are open-ended, manufactured or natural play materials that are moveable and without a dictated purpose, which may include anything from recycled tires and stumps, to car parts or pinecones [29],[30]. Literature exploring the impact of loose parts play suggests it may promote play participation and engagement, social negotiation, creativity, imagination, problem-solving, and improved physical activity [31]–[37]. Research has also suggested benefits of loose parts play include confidence, leadership, determination, resilience, and enabled risk-taking [38]. Loose parts offer the opportunity for adventurous, risky play, facilitate exploration and creativity, and allow children to direct their environments and play [34],[39]–[41]. Loose parts have been associated with risky play as they offer opportunity for climbing, swinging, and balancing, and encourage children to test their abilities, and negotiate and assess risk [34],[39],[41]. With evidence suggesting outdoor play and learning spaces rarely afford the opportunity for risky play [15], introducing loose parts may provide that opportunity.

Ecological approaches, or those that situate health across the context of complex, multi-level environments, from the micro to the macro level, are often employed in health promotion research, particularly in relation to physical activity [42]–[46]. Van Rooijen and Newstead (2017), drew upon ecological approaches to explore factors influencing professional attitudes toward risk-taking in childhood play [17]. They developed a model represented by concentric circles, with the practitioner centered, surrounded by increasingly larger levels of influence [17]. Their model includes how children are constructed (for example, as vulnerable and in need of protection); professionals' attitudes, beliefs, and values regarding risk; relationships with parents (requiring collaboration, trust, and communication); regulatory factors like playground restrictions, accountability, and liability; and finally broader cultural factors, including social, political, and environmental concerns [17]. They assert the complex interaction and interdependence of these factors results in significant ongoing conflict, negotiation, and contradictions experienced by childhood practitioners, and suggest future research is warranted to further explore their perspectives [17].

Expanding on existing literature, we explored the perspectives of early childhood educators who took part in the Physical Literacy in the Early Years (PLEY) project, an outdoor loose parts play intervention [47]. The purpose of this study was to explore early childhood educators' perspectives of risky play, and more specifically, risky play in the context of this outdoor loose parts play intervention, in alignment with the model proposed by van Rooijen and Newstead (2017) [17].

## 2. Materials and methods

### 2.1. Study design

This paper qualitatively describes educator perceptions of preschooler's risky outdoor play collected as part of a mixed methods intervention study, the Physical Literacy in the Early Years (PLEY) project (registered ID# ISRCTN14058106). The purpose of the PLEY project was to evaluate a loose parts intervention in regulated early childcare settings, including its impact on physical activity and outdoor play, and educator and parent perceptions. The PLEY intervention involved integrating loose parts (such as rope, milk crates, wood, tires, and buckets) into the outdoor play spaces of 11 childcare centres across Nova Scotia for periods between six and eight months. The project used a socioecological approach and the RE-AIM framework to explore multiple levels of influence and understand the impact of the intervention [46],[48]. Further details regarding the PLEY project intervention protocol are presented elsewhere [47].

Qualitative data were collected using Qualitative Description methodology. Qualitative Description is an exploratory methodology which, while less interpretive than other qualitative methodologies, is focused on describing the lived experiences of participants, from their perspectives [49],[50]. The emphasis on rich description, participant voice, and a tendency to remain close to the data, made Qualitative Description an ideal methodology for this study [49],[50].

### 2.2. Data collection and analysis

Early childhood educators from childcare centres involved in the PLEY intervention were invited to take part in focus groups. Fifteen focus groups took place, nine of which occurred three months following the intervention, and six of which occurred six months following the intervention. Each intervention site was represented in the focus groups and included early childhood educators from a variety of sites, with between three to five educators taking part in each focus group. Focus groups each lasted approximately 45–60 minutes and included questions regarding the intervention, active outdoor play, loose parts, and risk-taking. All focus groups were audio recorded and facilitated by a member of the research team and a notetaker.

Audio data from focus groups were transcribed verbatim, organized using Microsoft Word (version 16.16.3) and imported into QSR NVivo 11 for analysis. Thematic analysis was guided by the methods of Miles and Huberman [51] and Braun and Clarke [52]. Analysis was conducted by research staff and guided by senior members of the research team. Analysis began with research staff reviewing transcripts and identifying codes using open inductive coding. Frequent research team meetings facilitated the collaborative and iterative development of a codebook. Two coders coded each transcript early in analysis in order to facilitate consistency, and once consistent coding was established, remaining transcripts were coded by one member of the research team. Once transcripts were coded, a collaborative process was used to explore relationships between codes and identify trends across the data, in order to generate themes [51],[52]. Quality and rigour, including dependability, authenticity, and credibility, were facilitated using this collaborative and iterative process, in addition to using peer review, field notes, and memo-ing [53],[54]. Themes were then examined in consideration of ecological approaches and the model proposed by van Rooijen and Newstead (2017) [17].

## 3. Results

Four themes were identified through Qualitative Description and thematic analysis. The first theme describes how risky play with loose parts uncovers evolution in educator experience, perceptions, and practice; the second theme presents how educators' perceptions of risk are connected to institutions, systems, and discourses of safety; the third theme demonstrates how educators developed strategies to facilitate risky play with loose parts; and the final theme explores how educators perceive risky play as beneficial for children's healthy development.

### 3.1. Theme 1: Risky play with loose parts uncovers evolution in educator experience, perceptions, and practice

The first theme highlights how risky play and the loose parts intervention brought to light how educators' background, experience, and history contributed to their perceptions. As part of this theme, educators reflected on the historical and societal shifts that have occurred in the way children play. Participants reflected on their own experiences, including where and how they were raised, and played, and how their own experiences contributed to their feelings about risky play. One educator said, for example, *“we knew every inch of the woods all through our neighborhood and parents weren't with us, I'm not sure if that happens as much anymore”*. They related these perspectives of risky play to their work as early childhood educators, with one saying, *“when you've been in the business for so long you're ingrained of just keep them safe, make sure they're safe, the times are changing where risk is part of their play now”*, suggesting that attitudes around risky play have changed. One participant noted the loose parts intervention helped reveal this evolution in perspectives regarding risky play, saying *“perhaps before there wasn't enough risk taking, [...] I started to say to myself, I think we better stop saying, ‘be careful, be careful’ all the time”* noting the loose parts intervention as helpful in facilitating reflection on risky play.

Educators also reflected upon how they each have their own individual comfort level with risky play. Many participants described how they were more comfortable with risky play outside than inside. One said they were *“more free with the risk-taking outside”*, while another said, *“when they're outside they have the space to explore, they have the room to just run and jump”*. Another common perspective was that educators were mindful of the responsibility associated with caring for other people's children. One said, *“it's someone else's child and you want them to be in one piece at the end of the day”*. Several participants noted they were more comfortable with risky play with their own children, with one saying, *“I don't want other people's kids to get hurt, but like, I let my own kid do something and they get hurt I’d be like well you know that's my kid”*. Some participants reflected on how their own upbringing impacted their perspective on risky play, with one saying, *“as a child I did everything so I'm able to let them”*. Similarly, another participant said, *“we had strict rules of what happened in our house and it wasn't the risk play”*, and one considered, *“I wonder if my opinions would change if I was more of a risk taker as a child”*.

Educators also noted that they were progressively becoming more comfortable with risky play, and that this was facilitated by their participation in the outdoor loose parts play intervention. Educators relayed that systemic, environmental, and cultural shifts were happening regarding risky play, in association with the intervention. One said, for example, *“I think there's a change, there's an awareness that wasn't there before”*, while another said, *“we're seeing a shift within our own organization”*. Connecting to the earlier theme of confidence, a participant said, about children's risk-taking with loose parts, *“it's building the children's skills and confidence and then as that's happening, yours is also being built which means that there's this beautiful sort of mutual respect”*. Similarly, a participant said, *“the more educated you get about it [...] you feel more confident in yourself to let your children take the risks”*. Others discussed how their participation in the outdoor loose parts play intervention changed *“our mindset”* or “*changed my whole perception of risk”*. To sum, one educator said, *“we see the benefits of it now, not the scariness of it”*. Finally, some participants noted the impact of the intervention more broadly, saying, for example, *“I feel like being a part of this has allowed the company to loosen up a little bit”*, indicating that the outdoor loose parts play intervention contributed to changing perceptions of risky play institutionally. Educators recognized shifts in how risky play was perceived over time, that were contributed to by their own history, education, and experiences. The outdoor loose parts play intervention offered an opportunity for further learning and experiences, contributed to shifts in educator perceptions of risky play, and increased comfort in risky play.

### 3.2. Theme 2: Educator perceptions of risky play are tied to institutions and systems

Educators in this project also connected their perceptions of risky play to institutions and systems, and broad discourses around responsibility and safety. As part of this theme, educators articulated their fear of children getting hurt and being responsible for that injury. All participants discussed situations and experiences where they had experienced fear, worry, and anxiety associated with risky play. One said, *“as a teacher you just tend to feel responsible”*, a sentiment that was common among the educators in this project. Connecting to the above subtheme around individual comfort with risk, one participant said risky play had taken them *“out of my comfort zone for risk because I think of the worst scenario, not the best scenario”*. Others connected this to the above subthemes of perceptions of risky play changing over time, with one saying, *“I've been in the field for a long time [...] years ago it was ‘no you can't do that’ [...] it's a hard thing to let go of [...] nobody wants to see children be hurt but the more you let them do things you realize that yah it's risky but it's not usually that bad”*. One participant said that their role is about *“keeping everybody safe [...] I want to support the risky play, but at the same time we have to be careful”*, which was aligned with many participants who noted that there is a balance to be found between encouraging risk and ensuring safety.

Closely connected to the fears and worries that come with risky play, educators discussed their perceptions of rules, regulations, reporting, and responsibility. A common perception was that *“with the risk taking comes a lot of paperwork too”*. Many participants discussed the protocols and requirements associated with an injury. One said, *“you're writing the accident report in your head”* in regard to watching risky play take place, while another said, *“I mean every child's going to have a fall, every child is going at some point [...] you need a band-aid [...] but apparently now it's like any mark [...] it needs to be written up”*, noting a bit of fatigue with reporting, but also acknowledging that injuries may happen in risky play. Others acknowledged the obligations around risk to be important, with one saying, *“the policy is about accountability [...] our actions reflect on the centre”*. Other participants reflected on the importance of communication, saying, *“that's the way that administration would look at it, ‘how are you going to do this safely? Tell me why you want to do it’”*, highlighting the connection between their perception of risk and that of the administration.

### 3.3. Theme 3: Educators developed strategies to facilitate risky play with loose parts

Through this project, educators also reflected on the strategies they developed to support risky play and the use of loose parts. Observation was a key strategy noted by many of the participants in facilitating risky play. Educators discussed the importance of stepping back, staying close without interrupting, and letting children lead. One participant said, *“for me it was just the teaching strategy of letting her take the risk and letting her come to her own conclusions”*. Another said, *“If you're scared they're going to be scared, so just, you know, be nearby and let them try”*. Educators also often discussed how they speak to children engaging in risky play. One noted the importance of being *“more mindful of what I'm saying to them in those moments and if I need to say anything at all”* and the value of *“being supportive and not restrictive”*. Many discussed the strategy of asking questions, with one saying, *“I've been trying to steer away from ‘be careful’ and kind of phrase, like, more open-ended questions, like ‘what would happen if you step your foot there?’”*. Others discussed the importance of providing encouragement, with one saying, *“I wanted to let her be independent, let her be creative and definitely gave her words of encouragement, like ‘this is awesome [...] look at how you're balancing’”*. Finally, some participants discussed switching out with one another as educators, with one saying, *“maybe I'm going to step back because I know you're a little more comfortable and you can facilitate this”*, acknowledging again that individual educators will have varying comfort with risk.

Relatedly, educators mentioned the importance of communication in facilitating risky play and outdoor loose parts play. As mentioned, educators have individual experience, background, and comfort levels with risky play, and communicate with one another to ensure educators feel comfortable supervising each activity. Others discussed how risky play warranted communication when there were staff changes, with one saying, *“we've just recently had new staff that have come in so we kind of had to go through the whole process all over again explaining it and expectations but also like comfort levels and kind of where we're at with risk taking”*. Additionally, many participants discussed the importance of talking to parents about risky play. One said, *“we have lots of parent nights dedicated to it [...] we do try to really open up that as a talking point with our families so they're comfortable with things that we're doing with their children”*, while another said, *“talking it through with our parents and our families is really important because I don't want them to just walk in and think it's like [...] we're not being careful or thoughtful, that we're not being intentional, because we know the benefits”*. Others connected this more directly to the loose parts intervention, noting that *“it created a dialogue”* and *“gave us more tools to go and say this is why we're doing this [...] And how we're going to do this effectively”*, demonstrating the importance of clear communication with families regarding risky play and loose parts.

### 3.4. Theme 4: Educators perceive risky play with loose parts as beneficial for children's healthy development

The final theme describes how educators perceived risky play as beneficial for children's healthy development. Participants discussed their perceptions that children should take risks and want to take risks, and frequently discussed how risks come with rewards. One said, for example, *“they're up so high and they could fall down and hurt themselves, but there's so much more that they learn from it”*. Some described risky play as a natural and important part of healthy development, saying, *“it's part of growing up”*. Others noted how children wanted to engage in this type of play, with one saying, *“I think it was just, it was something they craved, like they kind of needed to explore it, they needed to see if they could”*. In addition, educators noted that risky play was perceived as *“thrilling to them”* and that it made *“outside time more exciting”*. Participants discussed the importance of boundary-pushing and thrill-seeking associated with risky play. One educator said, for example, *“they're pushing the boundaries to see how far they can go”*. Another participant said, *“it's also the forbidden fruit, my mother doesn't let me do that, but I can do that at the daycare”*, describing their perception of how children enjoyed risk-taking opportunities.

Educators also described their perception that risky play promoted problem-solving, social skills, and confidence. One participant noted that, *“the big improvement I think has been the children, you know who are really now taking more risks, and using a lot more problem solving”*. Participants also discussed how the children self-assessed risks and developed risk-management skills. One said, for example, *“the class independently decided that's too high, we should not jump from here”*, while another said, *“I think they know like where their limits are”*. Many educators discussed their perception that risky play promoted confidence. One said, for example, *“you really saw the child sort of push themselves out of their comfort zone”*, while another said, *“I think the more risk they take, the more confidence they have”*. This confidence was also associated with pride and was perceived to have long-term health benefits: *“it's a sense of pride, it's a sense of accomplishment and that's huge cause [...] that's what they see in the future and them taking risks in adulthood”*.

Participants also mentioned the value of risky play for supporting children's physical health and well-being. One participant noted that risk taking during outdoor loose parts play allowed children to *“really push themselves physically”*, and how, *“over time they're just taking bigger risks, using more muscles you know, using different muscles”*. By taking risks, children were developing critical fundamental movement skills, such as coordination and balance, and improving muscular strength and endurance: “*you can really see that their coordination is improving, their balance is improving”*; *“his muscle development was not quite there [...] and his parents had mentioned that he had come a long way [...] and he was enjoying the experience*.

Finally, there seemed to be consensus among participants that the loose parts intervention provided more opportunity for risky play. One educator said, *“I notice a lot more of that with the loose parts, like the risk-taking opportunities are awesome”*. Another added, about the loose parts intervention, *“it really added to our play and to their risks”*. Others discussed how children were more ready and prepared to take risks, with one saying, *“they were more eager to take risks [...] like after using these materials in different ways, they were more eager”*, and another agreeing, *“they're taking more risks than they would have, or higher risks than they would have”*. Regarding the loose parts, another participant said, *“it gives them the opportunity to scale their risk, depending on what they're comfortable with”*.

## 4. Discussion and conclusions

Through Qualitative Description, we identified four themes that can be analyzed using an ecological lens, specifically, the model developed by van Rooijen and Newstead (2017) to explore professional attitudes toward risky play [17]. The first theme highlights how risky play with loose parts contributes to an evolution in educator perceptions. This theme, emphasizing educators' perspectives regarding risky play, is well-aligned with van Rooijen and Newstead's (2017) assertion of the importance of professional attitudes toward risk [17]. The second theme, demonstrating how educator perceptions relate to systems and institutions supports van Rooijen and Newstead's (2017) suggestion of a layer of influence related to regulatory factors [17]. Our third theme, illustrating how educators developed strategies to facilitate risky play intersects with van Rooijen and Newstead's (2017) identification of the importance of relationships with parents, in that our work highlights the importance of communication [17]. Our fourth and final theme, regarding how educators perceive risky play as important for healthy child development aligns with multiple layers of van Rooijen and Newstead's (2017) model, including professional attitude toward risk, constructions of the child, and cultural factors [17]. Interestingly, each of our themes is further intertwined with both the inner layer, focused on the constructions of the child, and the outer layer, focused on cultural factors, of van Rooijen and Newstead's (2017) model, highlighting the interrelatedness of these levels. In each of our themes, it is evident that children are constructed both as in need of protection, but also as having agency, developing skills, and learning to negotiate risk. Cultural factors are emphasized throughout each theme as well, with our participants noting broad cultural shifts and evolution in play over time and discussing wide social and political discourse around safety and regulation. This study expands upon previous research by including the perceptions of educators on risky play within the context of a childcare-based outdoor loose parts play intervention.

Through this project, educators reflected on personal and professional development in association with risky play. Educators noted differences between themselves and their colleagues regarding risk taking, acknowledging the influence of their own upbringing and personal experiences. This is important, as educators' perceptions of outdoor play, specifically their beliefs and personal experiences, can influence their teaching and practice, as highlighted in van Rooijen and Newstead's (2017) model which devotes a layer of influence related to professionals' attitudes toward risk [17],[55]. Relatedly, educators identified a societal shift over time toward focusing on structured activities and increased supervision, resulting in decreased opportunity for risky play. This is aligned with other research that indicates children's participation in physical activity is shifting from unsupervised and unstructured outdoor and risky play to more structured and supervised activities [1],[4],[17],[56],[57]. This is further aligned with van Rooijen and Newstead's (2017) model which suggests the importance of both regulatory and cultural factors [17] This finding suggests childcare settings have a unique opportunity to provide children with an environment that allows them to explore and challenge themselves through risky play. Importantly, the fact that educators recognize this shift suggests that they understand their role in supporting quality play experiences.

Educators also reflected on how their participation in the outdoor loose parts play intervention improved their comfort with risky play. Through this intervention, educators had the opportunity to develop strategies that facilitated their engagement in risky play. Educators noted observation as a key strategy in supporting risky play. They also discussed the importance of communication regarding risky play and being mindful of how risk is framed. This aligns with strategies suggested when assessing risk taking during outdoor play, including considering both the child's and educators' comfort and abilities [58]. This is also aligned with how van Rooijen and Newstead's (2017) model suggests the importance of professionals' relationships with parents, which requires clear communication, collaboration, and trust [17]. Similarly, educators have emphasized the importance of understanding their own limits when it comes to assessing risk-taking during outdoor play and how this may sometimes limit children's participation in risky play [17],[58]. Together, these strategies highlight how educators have to negotiate risk taking in the childcare setting.

Through this project, educators also discussed how supporting risky play in the childcare setting comes with challenges and responsibilities. Although educators recognized the importance of risky play, they reflected on the need to balance risk and safety. Educators did express fear and anxiety associated with children injuring themselves through this type of play in the childcare setting. A growing culture of child safety with risk mitigation strategies and childcare centre regulations have impacted the way early childhood educators practice and support risky play; Educators voicing these concerns, and the fears of being perceived as irresponsible, is consistent with previous literature [15],[23]. This is closely connected to van Rooijen and Newstead's (2017) model's highlighting of regulatory factors as influential in how professionals negotiate risky play [17]. Although participating educators discussed their comfort with their own children engaging in risky play, they voiced how this level of comfort changes with other children due to centre policies and associated responsibilities. This finding suggests regulatory factors can hinder the way they support risk taking in the childcare environment. Educators also discussed the importance of communication with families about risky play, highlighting the need for additional strategies to support this type of communication. This finding is emphasized in the literature where communication with parents, and the need for training to support this dialogue, is critical in order for educators to practice in a way that is meaningful and beneficial for children [59]–[61], and again echoed in the layer of van Rooijen and Newstead's (2017) model emphasizing professional relationships with parents [17].

In this study, educators discussed how risk-taking in outdoor play is perceived as fun and thrilling, and how they believed this type of play was rewarding and important. This finding suggests that risky play is generally perceived positively and was encouraged in the participating child-care settings. This is an important finding, as educators' attitudes toward risky influences their practice [17],[23]. Likewise, educators discussed how risky play benefits important aspects of child development such as enhanced fundamental movement skills, improved problem-solving, social skills, and confidence, and enhanced self-assessment. These findings are consistent with the literature and suggest that risky play not only benefits children's development, but participating educators are able to connect risky play to pedagogy [1],[4],[6]. This implies that although perceptions of risky play may differ among educators, the idea of risky play is generally perceived positively by educators and is seen as a mechanism for developing various physical, cognitive, and socio-emotional benefits.

Educators also discussed how they perceived the loose parts intervention to have a positive impact on the way children engaged in risky play. Educators described how the intervention facilitated children's physical activity and physical literacy and contributed to the development of fundamental movement skills such as balance and coordination, while also improving confidence. This finding is aligned with literature that suggests loose parts diversify the play experience and afford more opportunity to engage in risky play, through physical activities like climbing and balancing, as well as through controlling their environments, and allowing children to explore [15],[34],[39]–[41].

Important connections can be drawn between the themes identified in this project and across the levels of the model developed by van Rooijen and Newstead (2017) [17]. Across the themes, educators reflected on their own background, upbringing, and experiences. These reflections offer valuable insight into how educators construct their perceptions regarding risk. Educator beliefs significantly influence how they practice in the early childcare setting, with research indicating the belief systems of educators are informed by their own personal experiences and serve as a mechanism for how they make teaching-related decisions [62]. In fact, research has found educators' beliefs and personality type to influence teaching practice more than factors such as centre resources [63]. Across themes, educators also discussed societal shifts around play, and their perceptions that over time and generationally, we have become more risk averse regarding play. In this project, educators noted that the loose parts intervention served as a facilitator for an alternative shift that supported the adoption of risky play. These cross-theme findings also cross the levels of van Rooijen and Newstead's (2017) model, highlighting the importance of the construction of children, professional attitudes toward risk, and cultural factors. Future research should continue to explore societal shifts in play, how perceptions of risk relate to those shifts, and how loose parts may contribute to these perceptions.

Through this project we were able to explore educators' perceptions of risky play in the context of an outdoor loose parts intervention. Educators seem to recognize and appreciate the value of healthy risk taking during outdoor play for child development, and how loose parts materials provide a mechanism for children to explore and challenge themselves. Educators discussed how they negotiate risky play, and shared strategies they use to ease. Future studies are needed to further explore, evaluate, and assess the strategies used by educators here, to determine their efficacy in facilitating risky play, and to identify additional strategies. Future research should also further explore how risky play is communicated in childcare settings: with and between educators and administration, with parents and families, and with children.

This project has several strengths and limitations. A strength of this project is its contribution to the literature by adding a qualitative exploration of early childhood educators' perceptions of outdoor risky play in the context of a loose parts intervention. An additional strength is its use of an ecological framework and the recently developed model of van Rooijen and Newstead (2017) [17]. A further strength is that the work was conducted by a diverse interdisciplinary team with expertise in the use of loose parts in childcare settings. An important limitation of this work is the limited diversity represented in the childcare centres. While childcares from across Nova Scotia were included, they are only representative of regulated childcare centres, and therefore do not include the perspectives of other forms of childcare, limiting the diversity of perspectives that might be included, especially those which may serve historically under-represented groups. Additionally, while educator perceptions make a valuable contribution to the literature, this study is limited by exclusively including educator perspectives, and not including the child perspective as well. Future research should explore child perspectives of risky play and the use of loose parts.

Through this study, we found that risky play with loose parts contributes to an ongoing evolution in educator perceptions, and that those educator perceptions are intertwined with institutional, systemic, and cultural influences. We also found that educators facilitate risky play with loose parts by learning and developing new strategies, and that they perceive risky play to be an important part of healthy child development. Sharing these insights with other early years stakeholders may provide a better understanding on the benefits of risky play and the associated contribution offered by loose parts materials.
